# Salud trasnacional: cooperación transpacífica entre Chile y Perú

**DOI:** 10.1590/S0104-59702025000100038

**Published:** 2025-09-15

**Authors:** Patricia Palma

**Affiliations:** i Profesora asociada, Universidad de Tarapacá. Arica – Chile. ppalma@academicos.uta.cl

En los últimos años, y particularly posterior a la epidemia de covid-19, la historia de la salud, y de las epidemias, ha cobrado un alto interés ya no sólo de historiadores de la medicina, lo cual se observa en el aumento de publicaciones de difusión, dirigidas a un público amplio (Ruiz-Domènec, 2020; Cueto, 2022; Armus, 2024). Sin embargo, los estudios sobre las repercusiones de las epidemias tienen una larga data en América Latina, y han sido numerosos los historiadores que han analizado las consecuencias y transformaciones que han generado para los Estados y la población. Si tomamos como ejemplo la revista *História, Ciências, Saúde - Manguinhos*, observaremos que desde 2000 a la fecha se han publicado casi cincuenta artículos que analizan algún tipo de epidemia en países como Argentina, Chile, Brasil o Perú. Asimismo, la experiencia de la covid-19 nos ha llevado a pensar cada vez más en la transmisión de enfermedades y el desarrollo de políticas de salud desde una perspectiva transnacional, pues por más esfuerzos que históricamente hayan puesto los gobiernos por cerrar sus fronteras terrestres, marítimas y aéreas, no estamos inmunes a las epidemias. Así, la experiencia actual e histórica indica que la cooperación, la comunicación y el desarrollo de políticas de salud en conjunto son claves para evitar la propagación de las enfermedades.

Desde una clasificación más clásica, el libro de Joshua Savala (2024) no es propiamente una historia de la salud en América Latina. Su objetivo es analizar diversas historias conectadas entre Chile y Perú, demostrando la existencia de un espacio compartido – el Océano Pacífico – que generó una circulación de ideas, personas y conocimientos entre estas dos naciones, que a su vez permitió la posibilidad de colaboración en diversos ámbitos sociales, laborales y económicos. El libro y cada uno de sus capítulos se construye a través de un análisis minucioso de información de diversos archivos y bibliotecas ubicadas en Chile, Perú, Países Bajos y los EEUU, incluyendo fuentes históricas de repositorios que escasamente han sido analizados, como el Archivo Histórico de la Facultad de Medicina de Lima.

Pese a no ser su enfoque principal, en las historias conectadas analizadas por el autor, la cooperación en la lucha contra las enfermedades y epidemias tiene un lugar importante. *Más allá de la guerra* es un novedoso y excelente trabajo para entender y complejizar nuestro conocimiento respecto a un sinfín de temas respecto a la historia entre Chile y Perú, de la historia del trabajo y los puertos como espacios multiculturales y homosociales, el rol de la policía y sus tempranos esfuerzos de vigilancia transnacional, la organización sindical y sus redes transpacíficas, entre muchos otros. Sin embargo, acá me enfocaré en el tema de salud transnacional.

El libro está compuesto por cinco capítulos que abordan los lazos transnacionales entre diversos actores, desde trabajadores no calificados (como pescadores) hasta profesionales (como médicos). En dos de sus capítulos (dos y tres) salud y enfermedad son preocupaciones centrales de trabajadores y autoridades. En el capítulo dos, “Género, masculinidad y sexualidad en el Pacífico”, el autor demuestra la preocupación de las autoridades sobre cómo la vida sexual de quienes habitaban y trabajaban en puertos y, en general, en el rubro marítimo estuvo estrechamente vinculada a las enfermedades venéreas. Así, en este capítulo se aborda cómo los Estados buscaron regular el trabajo sexual a partir de una serie de criterios médicos y sanitarios, en un espacio particularmente homosocial, en donde las relaciones sexuales no siempre se ajustaban a las normas del matrimonio.

El capítulo tres, “Cólera transnacional”, es un excelente ejemplo de cómo abordar la historia transnacional desde el punto de vista de la salud. Acá se van entrelazando historias de personajes que tuvieron que aprender e implementar políticas de salud para evitar la propagación del cólera, especialmente médicos. El autor demuestra que el cólera fue un aglutinante entre la comunidad de médicos de Chile y Perú, pese a un fuerte nacionalismo que existía al interior del gremio profesional. Los médicos habían tenido un rol clave en la Guerra del Pacífico (1879-1883), sin embargo, el temor a la propagación del cólera en 1887-1888 pudo unir a estas comunidades médicas más allá de resentimientos nacionales. En este capítulo se observa la importancia que tuvo la circulación de noticias en la implementación de medidas como las cuarentenas para barcos que regularmente viajaban entre ambos países.

Como *Más allá de la guerra* demuestra, el miedo a la propagación del cólera vigorizó la formación estatal tanto en Chile como en Perú, a partir de la creación de infraestructuras que incluían nuevas líneas telefónicas o lazaretos, así como la implementación de un reglamento de sanidad marítima. Finalmente, el autor, utilizando una perspectiva micro-histórica, analiza el caso del doctor peruano David Matto en Chile, enviado por su gobierno para aprender sobre la enfermedad. La historia de Matto da cuenta de redes de información, aprecio profesional, del aprendizaje de cómo enfrentar (o no) las epidemias, y la importancia de las redes de comunicación trasnacionales para poder hacer frente a éstas.

Como se mencionaba anteriormente, la diversidad temática hace que el libro sea de interés para un público amplio, pero también genera que el lector interesado en una temática en particular, en nuestro caso salud y medicina, quede con gusto a poco. Considero que *Más allá de la guerra* es una invitación a otros académicos, y especialmente a los más jóvenes, a analizar sus temas de estudios desde lo transnacional. Sin duda existieron muchos otros médicos y profesionales de la salud que, como David Matto, circularon, aprendieron y se cuestionaron sobre la mejor forma de enfrentar enfermedades que azotaron la región. Sabemos que trabajar con archivos de diversos países y ciudades no es sencillo, especialmente cuando existe escaso o nulo apoyo estatal o de las universidades para realizar viajes de investigación. La buena noticia es que la digitalización de bibliotecas y archivos crece cada día más y son un gran aliado para poder aventurarse a lo transnacional o comparativo (Palma, 2021).
